# Missed foot fractures in polytrauma patients: a retrospective cohort study

**DOI:** 10.1186/1754-9493-8-10

**Published:** 2014-02-25

**Authors:** Annette B Ahrberg, Benjamin Leimcke, Andreas H Tiemann, Christoph Josten, Johannes KM Fakler

**Affiliations:** 1 Department of Orthopedics, Traumatology and Plastic Surgery, University of Leipzig, Leipzig, Germany; 2 Department of Traumatology and Reconstructive Surgery, BG Hospital Bergmannstrost, Halle/Saale, Germany

**Keywords:** Polytrauma, Foot, Fracture, Missed injury, Trauma

## Abstract

**Background:**

Missed foot fractures are a known problem in the care of the traumatized patient. They do not usually have an influence on the survival, but on the long-term result and the quality of the patient’s life. The aim of this study is to find out how many of these fractures are overlooked in a Level I trauma center and what the consequences for the patients are hypothesing that patients with a delayed diagnosis will have worse clinical results.

**Methods:**

Forty-seven patients (7.3%) with foot fractures could be identified in 642 polytrauma patients, retrospectively. All patients were divided into two groups: early diagnosed fractures and delayed diagnosed fractures, the latter defined as diagnosed after Secondary Survey. Patients were evaluated according to the Hannover Outcome Score, the Short Form-36 Health Survey, the AOFAS Score and the Hannover Scoring System. The average follow-up was 5 years and 8 months. Reasons for overlooking a foot fracture were analyzed.

**Results:**

The foot fracture was early diagnosed in 26 (55.3%) patients, but delayed in 21 (44.7%). There were no significant differences in the mean stay in the hospital or in the ICU. The fractures that were most often missed were those of the cuboid or the metarsalia. The highest risk factor for a delayed diagnosis was a fracture already diagnosed on the same foot. In 52.4% of the delayed diagosed fractures, an operative therapy was necessary. There were no significant differences between the two groups in the clinical results.

**Conclusions:**

In summary, the results of this study show that foot injuries can be a safety problem for the patient and the examination of the feet in the trauma room has to be a compulsory part of the algorithm. Although the majority of delayed diagnosed foot fractures demonstrated comparable results to the immediately diagnosed fractures, approximately 10% might have benefited from an earlier diagnosis. Even if there were no significant differences in the clinical results, we have to be aware that missing a fracture in the foot can lead to worse results in the complete polytrauma care.

## Background

“An unrecognized fracture has the potential to convert the trauma surgeon’s finest effort into disaster [1].” Injuries of the foot do not have an influence on the polytrauma patient surviving the trauma, leave severe neurologic deficits like spine or head injuries do, or threaten our patients’ lives. And yet, they will have a crucial influence on how our patient will judge our work from trauma room to discharge. Eventually, they have a high influence on the patient’s outcome and degree of impairment [2]. In spite of this, foot injuries are often overlooked during Primary and Secondary Survey as well as in the further treatment, especially when there are more severe and potentially life threatening injuries to be dealt with. In a landmark article published in this journal, Pfeifer and Pape (2008) have found a rate of 1.3% to 39% of missed respectively delayed diagnosed injuries in polytrauma patients with foot and ankle injuries accounting for 8.1% to 25.1% of all musculoskeletal injuries [3]. Fractures of the foot have a significant influence on the clinical result of polytrauma patients [4-7]. Polytrauma care has reached a high standard in Northern America and Europe. Mean costs per polytrauma patient are $26,521 ($14,686-$43,000) [8]. Yet, we let “easy” fractures have such an influence on the result and we still overlook a high percentage of them. The purpose of this study was to define a delayed diagnosed fracture and see how it influences the overall outcome of the polytrauma patient and hyopthesing that patients with missed injuries will have worse clinical results.

## Methods

In a retrospective study, data of all patients who had been treated as polytrauma patients in our Level I Trauma Center between January 1, 2000 and December 31, 2004 were collected. In this period, 778 patients were documented as polytrauma patients, including patients who were transferred from other hospitals. Six hundred forty-two (82.5%) of these patients were polytrauma patients as defined by Tscherne in 1987 [9]. Fifty-four (8.5%) of them had one or more foot fractures. Patients with an age under 16 years at the time of the trauma or injuries of the foot other than fractures, e.g. burns or soft tissue injuries were excluded.

Forty-seven of fifty-five patients (87%) received complete diagnostics and therapy (endpoint: discharge from hospital) and were dismissed or died less than 24 hours after arrival. These patients were included for analysis of the quality and quantity of delayed diagnosed fractures and reasons for the delayed diagnosis. Six of the 47 patients had to be excluded from clinical follow-up due to other injuries, i.e. amputation of the lower extremity because of complex trauma or paraplegia. Four patients could not be contacted and seven refused to take part in the study. Thus, 30 of 47 (63.8%) patients could be clinically and radiologically examined in the follow-up, accounting for 3.9% of all the afore mentioned patients. A fracture was defined as “delayed diagnosed”, if it had been diagnosed anytime after the patient had left the trauma room and therefore after the Secondary Survey. Thus, a fracture could only be classified as “early diagnosed”, if it had been diagnosed in the trauma room. At follow-up, all patients filled in a questionnaire of the SF – 36 [10] As a complete Tertiary Survey had not been performed consistently in all patients, because of a lacking formal protocol, the time of diagnosis was analysed independently from the Tertiary Survey. The clinical and radiological outcome was evaluated by the American Orthopaedic Foot and Ankle Society Scores for the hindfoot and midfoot [11], by the Hannover Scoring System [12] and the Hannover Outcome Score [13]. X-rays of the feet (pa, lateral and oblique) were taken and supplemented by a Brodén view in case of a calcaneal fracture and a physical examination was performed by a trauma consultant. Epidemiologic data as well as information about the kind of trauma and the course of treatment were taken from the patients’ hospital charts.

For statistical analysis, we used parametric tests and if the data failed to fulfill scale levels non-parametric tests (Chi Square Test, T-Test, univariate ANOVA) were used. For all tests a significant correlation was assumed if the p value was <0.05. Data were collected by Microsoft Access and Excel 2003, statistical analysis was performed with SPSS Vers. 15.

## Results

The median age of the patients at the time of follow-up was 39.0 years with 15 being female (31.9%) and 32 male (68.1%). The average follow-up was 5 years and 8 months (± 1 year 7 months; 3 years 6 months to 7 years 9 months).

Traffic accidents by car (n = 14, 29.8%) and suicidal jumps (n = 13, 27.7%) caused most of the injuries followed by falls from great height (n = 11, 23.4%) and motorbike accidents (n = 4, 8.5%).

In 26 (55.3%) patients the foot fracture had been diagnosed early, but delayed in 21 (44.7%,) according to the above mentioned criteria. Median age of the patients with a delayed diagnosis was 44 years, compared to 38 years in patients with an early diagnosis. Regarding the consciouness (“oriented”, “somnolent” or “unconscious/anaesthetized”) when arriving in the trauma room, there were no significant differences between the two groups (p = 0.328). Alcohol and/or drug screening was positive in 40.4% of the delayed diagnosis and in 59.6% of the early diagnosis (p = 0.501). Table 1 gives an overview on the polytrauma scores and GCS of both groups.

**Table 1:** Polytrauma scores (Median)

	**Early (n = 28)**	**Delayed (n = 19)**	**Total (n = 47)**	**p**
ISS	29,0	27,0	27,0	0,130
GCS	13,3	15	14	0,305
PTS	19,5	19,0	19,0	0,303
NACA	3,0	3,0	3,0	0,546

ISS Injury Severity Score; GCS Glasgow Coma Scale; PTS Hannover Polytrauma Score, NACA.

The median length of stay in the hospital of all patients was 31 days, with 6 days in ICU. Median stay in ICU was 8.0 days for early diagnosed patients and 4.0 days for delayed diagnosed with no significant difference.

In total, 153 different fractures could be diagnosed in 54 patients. Eleven patients suffered fractures in both feet, with 58 injured feet being analyzed. Fractures of the left foot (n = 37; 63.8%) were more frequent than of the right foot (n = 21; 36.2%). In 12 cases (20.7%) fractures were classified as open fractures.

In 60.4% of the patients, 44 accompanying fractures of the lower extremity had been diagnosed: 13 fractures of the ankle (22.4%), 17 diaphyseal fractures (29.3%), 4 fractures of the knee (6.9%) and 14 fractures of the femur (24.1%). Table 2 gives an overview on the foot fractures.

**Table 2:** Foot fractures in total numbers and percentages

**Bone**	**Number**	**Percentage [%]**
Calcaneus	31	20.2
Talus	17	11.1
Metatarsalia	64	41.8
Navicular	15	9.8
Cuboid	10	6.5
Cuneiformia	14	9.2
Phanlages	2	1.3

A conservative therapy was done in 13.5% of the early diagnosed fractures and 19.1% of the delayed diagnosed fractures, whereas 86.5% of the early diagnosed fractures and 80.9% of the delayed diagnosed fractures were treated operatively.

A closed reduction was possible in 18.9% of the early diagnosed cases compared to 14.3% in the delayed diagnosed cases.

### Analysis of the delayed diagnosed fractures

Forty out of 153 fractures in 21 patients had been delayed diagnosed, counting for 26.1% of all fractures in 38.9% of 54 patients. A Chopart dislocation had been delayed diagnosed in 2 cases (16.7%), 22 (55%) of the delayed diagnosed fractures were not dislocated, and 18 (45%) were dislocated; none of the fractures were open. The diagnosis of the delayed diagnosed fractures had a median time of 11 days (0–166 days ± 35.2 days). In three patients (14.3%), the diagnosis had been made within 24 hours after admission, 11 (52.4%) within 10 days, and 19 patients were diagnosed during their stay in the hospital (90.5%). Six cases (28.6%) had been referred from other hospitals (all of them on the day of trauma), but only in half of these cases the fractures had been diagnosed immediately (Table 3).

**Table 3:** Foot fractures in total numbers and percentages in the delayed diagosed cases

	**Total**	**Delayed**
Calcaneus	31 (20.2%)	6 (19,4%)
Talus	17 (11.1%)	5 (29,4%)
Naviculare	15 (9.8%)	5 (33,3%)
Cuboid	10 (6.5%)	4 (40,4%)
Cuneiformia	14 (9.2%)	4 (28,8%)
Metatarsalia	64 (41.8%)	16 (35%)
Phalanges	2 (1.3%)	0

### Reasons for a delayed diagnosis

The following reasons for a delayed diagnosis could be identified:

• Diagnostics not performed n = 7 (33.3%)

• Insufficient quality of X-rays n = 6 (28.5%)

• Overlooked in an X-ray n = 5 (23.9%)

Reasons for not making the correct diagnosis were:

• Fracture not seen in CT or X-rays n = 14 (66.7%)

• No diagnostics in spite of clinical symptoms n = 2 (9.5%)

• Absence of clinical symptoms n = 4 (19.1%)

• Interruption of diagnostis because of circulatory problems n = 1 (4.7)

The fractures were finally diagnosed because of:

• Pain, Swelling n = 5 (23.8%)

• Preoperative CT n = 4 (19.1%)

• Preoperative X-ray n = 2 (9.5%)

• Postoperative X-ray n = 6 (28.6%)

The diagnosis of a fracture in a preoperative CT or X-ray or in a postoperative X-ray was possible, since more than one fracture could occur in both feet.

In 27 (67.5%) delayed diagnosed fractures, there were other fractures on the same foot, the contralateral foot or the distal extremitiy below the knee. In 12 cases, a fracture was delayed diagnosed when another fracture had been diagnosed on the same foot before. The following risk factors for overlooking a fracture could be identified:

• Fractures of the same foot (12 fractures)

• Fractures of the contralateral foot (7 fractures)

• Fractures of the same calf (6 cases)

• Fractures of the contralateral calf (4 cases)

Regarding the complexity of the foot trauma according to Zwipp, there was a tendency to delayed diagnosis in trauma grade 2 or higher, even though it was not significant (*p* = *0.145*) [2].

### Consequences of a delayed diagnosis

In 11 cases (52.4%), an operative therapy of the delayed diagnosed fracture was necessary. Retrospectively, the delayed diagnosis did not change the way of treatment in 19 patients (90.4%). In one case (4.8%) an amputation of the leg below the knee had become necessary due to severe soft tissue damage without the missed fracture influencing the decision. In another case, the fracture had already healed with a satisfactory result. This was the only case in which an immediate diagnosis would have resulted in a different method of treatment.

Regarding soft tissue problems, infections or other postoperative complications there were no significant differences between the two groups (p < 0,05).

### Clinical scores

**Figure 1:**
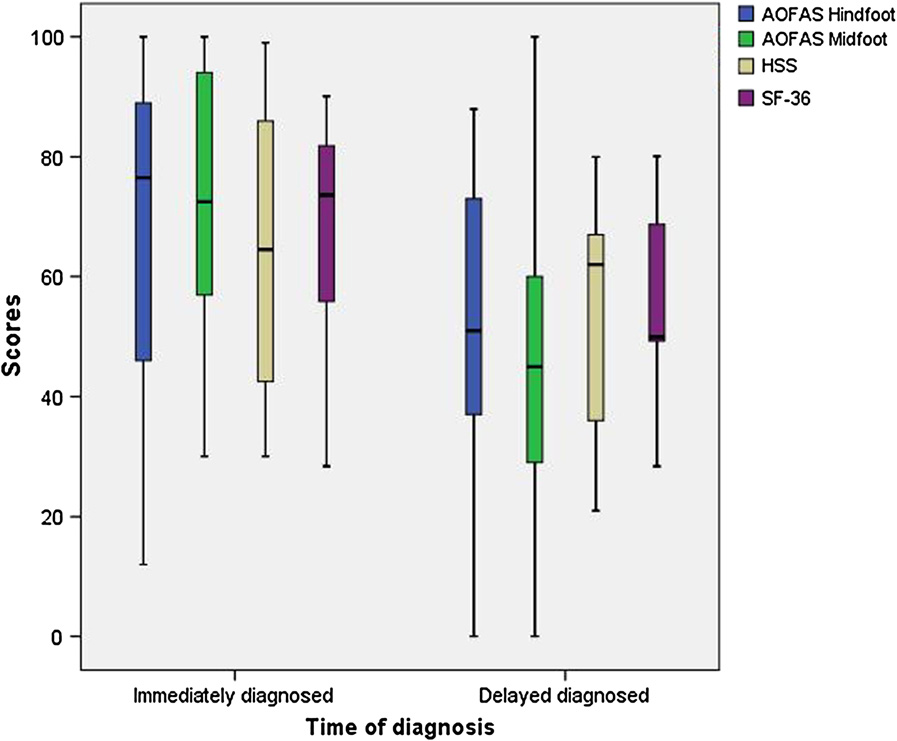
Boxplots of the clinical results.

Comparing the clinical scores, there were no sigificant differences between the two groups except for the AOFAS Midfoot Score, even though group ID had higher medians in all scores (Table 4; Figure 1).

**Table 4:** Clinical results of both groups

		**Total**	**Early diagnosed**	**Delayed diagnosed**	**P**
AOFAS Hindfoot	Median	69.0	76.5	51.0	0.244
	*n*	22	16	6	
	SD	28.2	26.6	31.7	
AOFAS Midfoot	Median	67.0	**72.5**	**45.0**	**0.045**
	*n*	22	16	6	
	SD	27.9	22.7	33.3	
HSS	Median	64.0	64.5	62.0	0.380
	*n*	22	16	6	
	SD	23.1	23.6	21.9	
SF-36	Median	60.1	63.7	51.7	0.300
	*n*	35	23	12	
	SD	19.7	20.4	17.9	
HS	Median	65.0	70.5	64.0	0.134
	*n*	29	19	10	
	SD	20.3	20.1	18.1	

Table 4 compares the medians and standard deviations of both groups. Significant results (AOFAS Midfoot) in boldface.

There were no significant correlations between the time of diagnosis in days and the single scores. Linear regression analysis showed that HSS and AOFAS Scores were negatively influenced by a higher number of days between trauma and diagnosis, unsatisfactory operative results, complexity of the foot trauma and need for changing the profession.

## Discussion

Foot fractures are typical injuries after falls from a great height. The percentage of 51.1% in this study is not surprising [6]. Contrary to the studies of Rizoli et al. (1994) or Pfeifer and Pape (2008), a higher severity of trauma (ISS, PTS) or a decreased consciousness (intubation, alcohol/drugs, GCS < 10) were not associated with a higher rate of delayed diagnosed foot fractures [3,14]. The results of this study confirm those by Robertson (1996) and Brooks et al. (2004) [1,15]. The level of consciousness as well as the severity of the trauma seem to be of minor importance in that matter. Foot fractures occur in 3.7% to 8.7% of polytrauma patients, even up to 13.6% in ISS ≥ 16 [6,16]. Therefore, the rate of 6.9% is within that range.

In 66.7% of the delayed diagnosed cases, fractures had been missed in CTs and X-rays. Thus, the highest risk factor for overlooking a fracture had nothing to do with circulatory problems, but simply not recognizing it. The risk was especially high if there was another fracture on the same or contralateral foot. In conclusion, the diagnosis of one fracture in the foot must make the examiner more aware of possible further fractures. It is noteworthy that, at least retrospectively, there were no clinical symptoms, e.g. hematoma or swelling, of the fractures in 19.1%. Robertson also found a rate of 23% with no indication to suggest injury [1]. This underlines the importance of performing a Tertiary Survey including a diligent examination of the feet.

Most of the delayed diagnosed fractures were not or only slightly displaced. Retrospectively, in 90.4% of the cases an earlier diagnosis would not have changed the treatment. This explains the non-significant difference in the clinical scores to the immediately diagnosed cases. Patients with a delayed diagnosis did not spend longer time in the hospital or in the ICU, so the delayed diagnosis of a foot fracture does not influence this factor.

In this study, the foot fracture did not significantly influence the outcome of the patients. Yet, it is known that polytrauma patients with a foot injury have worse outcomes than those without [4,6]. So even if there were no significant differences in the clinical results, the delayed diagnosis of a foot fracture increases the risk of a worse overall result and therefore is a safety issue for the patient. Especially if considering that the patient’s knowledge about a missed fracture influences his subjective result [17,18]. Table 5 gives an overview on various studies on missed injuries in polytrauma patients with regard on fractures of the foot.

**Table 5:** Overview on studies on delayed diagnoses in multiple injured patients with regard to the feet

**Authors**	**n**	**Delayed diagnoses total [%]**	**Foot fractures in delayed diagnosis [%]**	**Prevalence of delayed diagnoses in foot fractures [%]**
Chan et al. (1980) [19]	327	11.9	20.5	2.5
Born et al. (1989) [20]	1006	3	10.3	0.7
Juhl et al. (1990) [21]	783	2.2	10.3	1.5
Ward and Nunley (1991) [22]	111	18	7.2	2.7
Laasonen and Kivioja (1991) [23]	340	4.2	26.7	3.5
Metak et al. (1994) [24]	323	12.4	20	2.5
Rizoloi et al. (1994) [14]	432	13.6	6.8	0.9
Kremli et al. (1996) [25]	638	6	13.8	1.7
Robertson et al. (1996) [1]	3996	1.4	5.7	0.1
Janjua (1998) [26]	206	65	-	-
Guly (2001) [27]	934	100	6.4	6.6
Houshian et al. (2002) [28]	786	8.1	5.5	0.9
Vles et al. (2003) [29]	3879	1.3	12.2	0.2
Sharma et al. (2006) [30]	163	57.6	1.8	0.01
Wei et al. (2006) [31]	2407	3.7	7.6	0.3
This study	778	-	100%	2.3%

## Conclusions

In summary, the results of this study show that with 40% of the delayed diagnosed fractures foot injuries can be a safety problem for the patient. They confirm that the examination of the feet in the trauma room must be performed diligently within the Secondary Survey. It may not be the primary examination when treating a polytrauma patient, but has to be a compulsory part of the algorithm. The examination of the feet has to be part of a standardized Tertiary survey protocol. The examination should be repeated in the ICU and in the ward, a rule that should not only be applied on the feet. The diagnosis of a fracture in the foot should make the examiner look for further fractures and although the majority of delayed diagnosed foot fractures demonstrated comparable results to the immediately diagnosed fractures, approximately 10% might have benefited from an immediate or earlier diagnosis. Even if there were no significant differences in the clinical results, we have to be aware that misssing a fracture in the foot can lead to worse results in the complete polytrauma care.

## Competing interests

The authors declare that they have no competing interests.

## Authors’ contributions

ABA performed the statistical analysis and drafted the manuscript. BL participated in the design of the study, examined the patients and performed the statistical analysis. AHT conceived of the study and supervised the examinations. CJ gave final approval of the version to be published. JKMF helped to draft the manuscript and gave final approval of the version to be published. All authors read and approved the final manuscript.

## References

[B1] Robertson R Mattox R Collins T Parks-Miller C Eidt J Cone J Missed injuries in a rural area trauma center Am J Surg 1996 172 564 567 discussion 567–568 10.1016/S0002-9610(96)00247-4 8942564

[B2] Rammelt S Biewener A Grass R Zwipp H [Foot injuries in the polytrauma patient] Unfallchirurg 2005 108 858 865 10.1007/s00113-005-0993-1 16133285

[B3] Pfeifer R Pape H-C Missed injuries in trauma patients: a literature review Patient Saf Surg 2008 2 20 10.1186/1754-9493-2-20 18721480 PMC2553050

[B4] Tran T Thordarson D Functional outcome of multiply injured patients with associated foot injury Foot Ankle Int 2002 23 340 343 11991481 10.1177/107110070202300409

[B5] Turchin DC Schemitsch EH McKee MD Waddell JP Do foot injuries significantly affect the functional outcome of multiply injured patients? J Orthop Trauma 1999 13 1 4 10.1097/00005131-199901000-00001 9892116

[B6] Probst C Richter M Lefering R Frink M Gaulke R Krettek C Hildebrand F Incidence and significance of injuries to the foot and ankle in polytrauma patients–an analysis of the trauma registry of DGU Injury 2010 41 210 215 10.1016/j.injury.2009.10.009 19889412

[B7] Stiegelmar R McKee MD Waddell JP Schemitsch EH Outcome of foot injuries in multiply injured patients Orthop. Clin. North Am 2001 32 193 204 x 10.1016/S0030-5898(05)70203-0 11465129

[B8] Willenberg L Curtis K Taylor C Jan S Glass P Myburgh J The variation of acute treatment costs of trauma in high-income countries BMC Health Serv Res 2012 12 267 10.1186/1472-6963-12-267 22909225 PMC3523961

[B9] Tscherne H Regel G Sturm JA Friedl HP [Degree of severity and priorities in multiple injuries] Chirurg 1987 58 631 640 3677879

[B10] Bullinger M Kirchberger I Ware J The german SF-36 health survey translation and psychometric testing of a generic instrument for the assessment of health-related quality of life J Public Health 1995 3 21 36 10.1007/BF02959944

[B11] Kitaoka HB Alexander IJ Adelaar RS Nunley JA Myerson MS Sanders M Clinical rating systems for the ankle-hindfoot, midfoot, hallux, and lesser toes Foot Ankle Int 1994 15 349 353 10.1177/107110079401500701 7951968

[B12] Richter M Causes, treament and prevention of fractures of the midfoot - clinical, technical and experimental studies PhD Thesis Medizinische Hochschule Hannover 2001

[B13] Held C Subtalar fusion after conservatively or operatively treated intraarticular calcaneal fractures - longterm results PhD Thesis Medizinische Hochschule Hannover 1999

[B14] Rizoli SB Boulanger BR McLellan BA Sharkey PW Injuries missed during initial assessment of blunt trauma patients Accid Anal Prev 1994 26 681 686 10.1016/0001-4575(94)90030-2 7999213

[B15] Brooks A Holroyd B Riley B Missed injury in major trauma patients Injury 2004 35 407 410 10.1016/S0020-1383(03)00219-5 15037376

[B16] Seekamp A Regel G Bauch S Takacs J Tscherne H [Long-term results of therapy of polytrauma patients with special reference to serial fractures of the lower extremity] Unfallchirurg 1994 97 57 63 8153642

[B17] Ott R Holzer U Spitzenpfeil E Kastl S Rupprecht H Hennig FF [Quality of life after survival of severe trauma] Unfallchirurg 1996 99 267 274 8658206

[B18] MacKenzie EJ Shapiro S Smith RT Siegel JH Moody M Pitt A Factors influencing return to work following hospitalization for traumatic injury Am J Public Health 1987 77 329 334 10.2105/AJPH.77.3.329 3812840 PMC1646910

[B19] Chan RN Ainscow D Sikorski JM Diagnostic failures in the multiple injured J Trauma 1980 20 684 687 10.1097/00005373-198008000-00009 7401210

[B20] Born CT Ross SE Iannacone WM Schwab CW DeLong WG Delayed identification of skeletal injury in multisystem trauma: the “missed” fracture J Trauma 1989 29 1643 1646 10.1097/00005373-198912000-00010 2593194

[B21] Juhl M Møller-Madsen B Jensen J Missed injuries in an orthopaedic department Injury 1990 21 110 112 10.1016/0020-1383(90)90067-5 2351463

[B22] Ward WG Nunley JA Occult orthopaedic trauma in the multiply injured patient J Orthop Trauma 1991 5 308 312 10.1097/00005131-199109000-00009 1941313

[B23] Laasonen EM Kivioja A Delayed diagnosis of extremity injuries in patients with multiple injuries J Trauma 1991 31 257 260 10.1097/00005373-199131020-00019 1994088

[B24] Metak G Scherer MA Dannöhl C [Missed injuries of the musculoskeletal system in multiple trauma–a retrospective study] Zentralbl Chir 1994 119 88 94 8165885

[B25] Kremli MK Missed musculoskeletal injuries in a university hospital in Riyadh: types of missed injuries and responsible factors Injury 1996 27 503 506 10.1016/0020-1383(96)00044-7 8977838

[B26] Janjua KJ Sugrue M Deane SA Prospective evaluation of early missed injuries and the role of tertiary trauma survey J Trauma 1998 44 1000 1006 discussion 1006–1007 10.1097/00005373-199806000-00012 9637155

[B27] Guly HR Diagnostic errors in an accident and emergency department Emerg Med J 2001 18 263 269 10.1136/emj.18.4.263 11435359 PMC1725632

[B28] Houshian S Larsen MS Holm C Missed injuries in a level I trauma center J Trauma 2002 52 715 719 10.1097/00005373-200204000-00018 11956389

[B29] Vles WJ Veen EJ Roukema JA Meeuwis JD Leenen LPH Consequences of delayed diagnoses in trauma patients: a prospective study J Am Coll Surg 2003 197 596 602 10.1016/S1072-7515(03)00601-X 14522329

[B30] Sharma BR Gupta M Harish D Singh VP Missed diagnoses in trauma patients vis-à-vis significance of autopsy Injury 2005 36 976 983 10.1016/j.injury.2004.09.025 16005004

[B31] Wei C-J Tsai W-C Tiu C-M Wu H-T Chiou H-J Chang C-Y Systematic analysis of missed extremity fractures in emergency radiology Acta Radiol 2006 47 710 717 10.1080/02841850600806340 16950710

